# Study of the Portevin-Le Chatelier (PLC) Characteristics of a 5083 Aluminum Alloy Sheet in Two Heat Treatment States

**DOI:** 10.3390/ma11091533

**Published:** 2018-08-25

**Authors:** Ni Tian, Guangdong Wang, Yiran Zhou, Kun Liu, Gang Zhao, Liang Zuo

**Affiliations:** 1 Key Laboratory for Anisotropy and Texture of Materials (Ministry of Education), Northeastern University, No. 3–11, Wenhua road, Heping district, Shenyang 110819, China; zhaog@mail.neu.edu.cn (G.Z.); lzuo@mail.neu.edu.cn (L.Z.); 2 School of Materials Science & Engineering, Northeastern University, No. 3–11, Wenhua road, Heping district, Shenyang 110819, China; w15041838726@163.com (G.W.); zhouyr@mail.neu.edu.cn (Y.Z.); 3 Department of Applied Science, University of Quebec at Chicoutimi, 555 Boulevard de l’Université, Chicoutimi, QC G7H2B1, Canada; kun.liu@uqac.ca

**Keywords:** 5083 aluminum alloy, PLC characteristic, annealed, quenched, strain rate

## Abstract

In the present work, the role of Mg atoms in the form of either Mg clusters or β phase on the moving dislocations in 5083 aluminum alloy sheet were investigated by comparing the plastic flow behavior and Portevin-Le Chatelier (PLC) character in annealed and quenched conditions. It is found that the tensile strength of quenched sheets at different strain rates is slightly higher than those under annealed condition while the yield strength at both conditions is similar. In annealed sheets, the yield plateau was clearly observed at all tested strain rates with a strain less than 0.012, and its width increased with the increasing strain rate. However, no yield plateau was observed in quenched sheets. On the other hand, the characters of PLC are greatly varied with applied conditions and strain rate. Generally, annealed sheets have a higher waiting time, but lower critical strain/stress at lower strain rate (~1 × 10^−4^ s^−1^), but they are similar at a higher strain rate (1 × 10^−2^ s^−1^). However, the falling time at both annealed and quenched conditions are almost the same at tested strain rates.

## 1. Introduction

The 5083 aluminum alloy, which has high corrosion resistance, excellent weldability and good strength, is widely used in shipbuilding, automotive, aerospace and industrial construction [1,2,3]. However, the phenomenon of serrated yielding or serrated flow, also known as the Portevin-Le Chatelier (PLC) effect, is easy to observe in the process of 5083 aluminum alloy [4,5,6]. On one hand, the PLC effect makes the surfaces of the products rough and reduces the surface quality. On the other hand, the PLC effect also reduces the ductility and affects the mechanical properties of the material, greatly reducing the formability of materials [7,8]. Dynamic strain aging (DSA) is considered to be the underlying mechanism that causes the PLC effect because of the interaction of mobile dislocations and diffusing solute atoms [9,10,11,12,13,14,15].

Factors that influence the results of the PLC effect include external parameters (strain, strain rate, temperature, hardness of stretching machine, specimen geometry, etc.) and internal parameters (alloy composition, crystal or polycrystalline, lattice, grain size, solute atom concentration, movable dislocation density, barrier type, etc.) [16]. Recently, several studies have been conducted using different methods on the serration behavior or PLC effect of 5083 aluminum alloy with regard to external parameters, such as strain rate [4,17], strain [17,18], temperature [18], and specimen geometry [19]. In addition, some studies focused on grain size [18,20] and movable dislocation density [18] regarding the internal parameters of influence of the PLC effect of 5083 aluminum alloy. Those research results [4,17,18,19,20] showed that the 5083 aluminum alloy is susceptible to dynamic strain aging and that Mg atom has a direct relationship with the PLC effect. The solubility of Mg atoms at room temperature is less than 1% by weight according to the Al-Mg binary phase diagram [21], whereas the content of Mg in 5083 aluminum alloy is 4.0–4.9 (wt.%). Some of the excess Mg atoms exist in the matrix as supersaturated atoms, and the other Mg atoms are in the form of β phase (Mg_5_Al_8_). However, little work can be found on the effect of Mg atom configuration on the serration behavior of PLC effect in 5083 series aluminum alloy. In 2004, Wen et al. [22] found that the precipitation of Mg atoms from a solid solution decreases the serration intensity in both Lüders straining and the subsequent PLC straining in DC AA5182 aluminum alloy under recrystallized, recovered and cold-rolled conditions. In 2013, Sarkar et al. [8] found that excess vacancies in the Al-2.5% Mg alloy after water quenched at 400 °C led to a decrease in the strain rate sensitivity and an increase in the number of stress drop occurrences per unit time, and the domain size and dislocation density have no appreciable effect on the PLC effect. 

In this paper, the form of the Mg atoms in 5083 aluminum alloy sheets was changed by controlling the cooling method after heat treatment, and then the influence of the Mg atoms in the forms of solute atoms and β phase on the movement of dislocation and the PLC effect of 5083 aluminum alloy sheets were investigated.

## 2. Materials and Methods 

**Figure 1:**
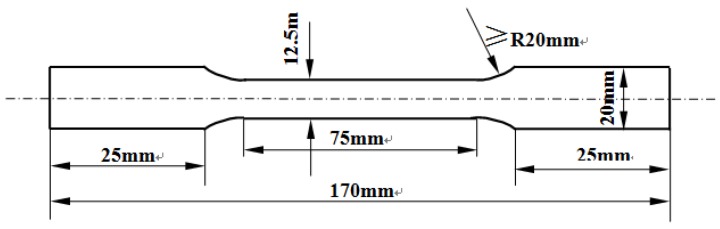
Size of the tensile specimens with a thickness of 2.8 mm.

The 5083 aluminum alloy sheet with a thickness of 2.8 mm used in this work was rolled from a plate with a thickness of 8 mm, and the composition of the alloy is as follows (wt. %): 4.54 Mg, 0.72 Mn, 0.28 Fe, 0.21 Si, 0.20 Zn, <0.1 others, and Al balance. The tensile specimens with gauge length, width, and thickness of 75, 12.5, and 2.8 mm, respectively, were cut from the alloy sheet along the rolling direction, the specific size of tensile specimens is shown in Figure 1. Half of the tensile specimens were annealed at 450 °C for 1 h in a resistance furnace and then allowed to cool in the furnace for 23 h (namely annealed), and the other half were solutionized at 450 °C for 1 h in a salt-bath furnace and then water quenched as the solution treatment (namely, quenched).

The tensile experiments were conducted using a Shimadzu AG-X100 electronic universal testing machine (Shimadzu Corporation, Kyoto, Japan) at room temperature for strain rates of 1 × 10^−5^ s^−1^, 1 × 10^−4^ s^−1^, 1 × 10^−3^ s^−1^, and 1 × 10^−2^ s^−1^. The data acquisition frequency of the machine is 100 Hz, the load accuracy is 100 N, the stress and the strain was measured by force transducer and extensometer, respectively. Then the typical curve was selected out of three repeat specimens in each section. The grain size of 5083 aluminum alloy sheet was observed using an Olympus GX71 optical microscope (Olympus Corporation, Tokyo, Japan). The anode film of a metallographic specimen was treated under 50 V for 50 s using an anode film solution with the following composition: 24% alcohol, 74% deionized water, 1% HF and 1% HBF_4_. The configuration of second-phase particles was determined using an Olympus LEXT-3100 laser scanning confocal microscope (LSCM) (Olympus Corporation, Tokyo, Japan) and a JEOL JEM-2100F transmission electron microscope (TEM, JEOL Ltd., Tokyo, Japan) at an accelerating voltage of 200 kV. Specimens for TEM observation were cut from the tensile specimen before and after tensile deformation, thinned to approximately 90 μm, and then electropolished in a twin-jet polishing unit operating at 15 V and −25 °C using a 25% nitric acid and 75% methanol solution until (MTP-1A magnetic twin-jet electropolishing, Shanghai Jiaoda electromechanical technology development co. Ltd, Shanghai, China) perforation occurred.

**Figure 2:**
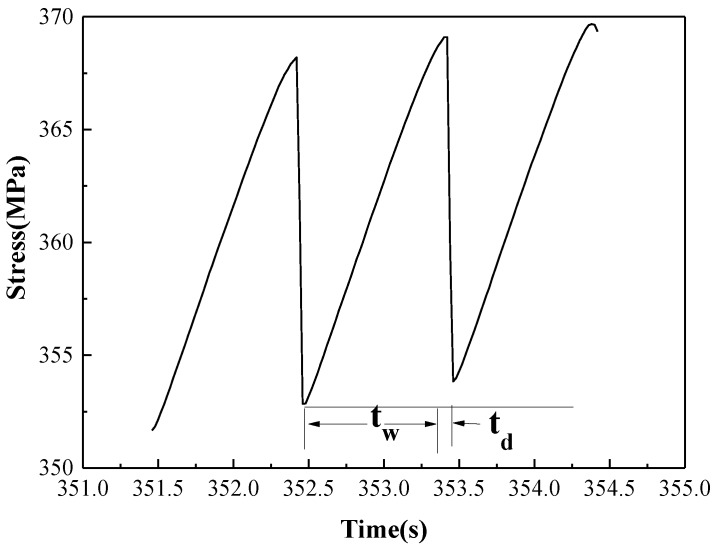
Schematic diagram of the waiting time (t_w_) and falling time (t_d_).

In addition, three feature quantities were used in this paper to characterize and measure the PLC effect: waiting time (t_w_), falling time (t_d_), and critical stress (ε_c_), which are illustrated in Figure 2. The waiting time (t_w_) is the time duration the stress increase, and this parameter corresponds to the time of dislocation temporally arrested at local obstacles. The falling time (t_d_) is the time duration the stress drop, and this parameter corresponds to the time of the mobile dislocation unpinned in an obstacle. The critical strain (ε_c_) and the critical stress (σ_c_) are the strain value and stress value when jerky flow begins to appear in the stress-strain curve.

## 3. Results and Discussion

### 3.1. Microstructures of Two Heat Treatment Conditions of 5083 Aluminum Alloy Sheets

**Figure 3:**
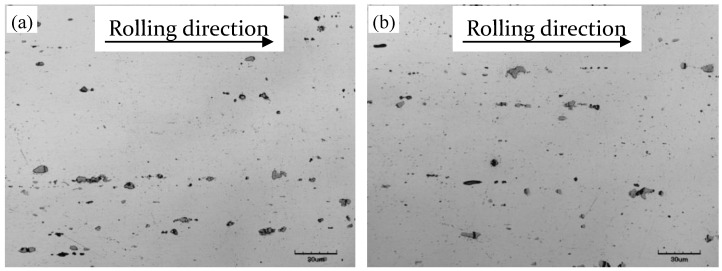
Optical micrograph of (**a**) annealed and (**b**) quenched 5083 aluminum alloy sheets.

The optical micrographs of 5083 aluminum alloy under two heat treatment conditions are shown in Figure 3. A large number of second-phase particles are observed in both annealed and quenched alloys matrices, which were distributed as broken chains along the rolling direction. The number and size of those second-phase particles at micro size have no obvious difference between annealed and quenched alloy sheets. The large gray particles in the matrix are the remaining constituent (FeMn)Al_6_, the crystallization temperature of (FeMn)Al_6_ and other constituents distributed along the rolling direction of the 5083 aluminum alloy sheet is over 600°C, as reported by Ratchev [23], and annealing and quenching at 450 °C have no effect on those second-phase particles.

**Figure 4:**
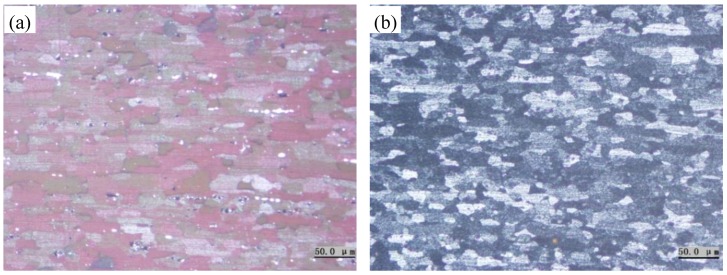
The grains of 5083 aluminum alloy sheets in two heat treatments: (**a**) annealed and (**b**) quenched.

Figure 4 shows the grains of 5083 aluminum alloy sheets under two heat treatment conditions. The grains of the annealed and quenched alloy sheets are found to be almost the same size, and both tend to be nearly equiaxed in shape.

**Figure 5:**
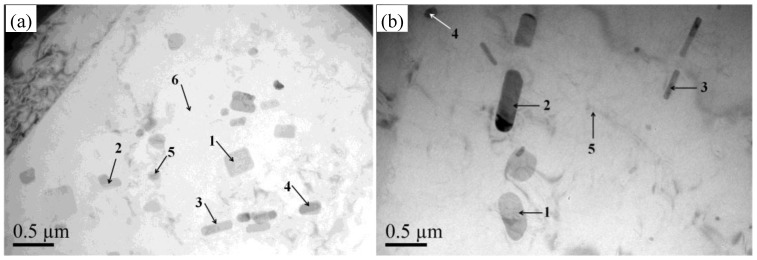
TEM images of (**a**) annealed and (**b**) quenched 5083 aluminum alloy sheets before tensile deformation.

Figure 5 shows transmission electron micrograph images of 5083 aluminum alloy sheets at annealed and quenched conditions before tensile deformation. Many second-phase particles are observed in the matrix of both annealed and quenched 5083 aluminum sheets. The number of particles in the annealed alloy sheet is obviously much higher than that in the quenched alloy sheet, and its distribution is also more dispersed than that of the quenched alloy. From the conducted spectrum analysis of the marked points shown in Figure 5a,b, the irregular polygon or rod products marked 1, 2, 3, and 4 in Figure 4a were identified as Mn-containing dispersoids, and the ultrafine spherical particle with a size within 100 nm was the Mg-containing β phase (marked 5 in Figure 5a), which occurs as a result of precipitation from the matrix during the slow cooling process of annealing. However, all of the strip or rod products shown in Figure 5b were Mn-containing dispersoids, with no Mg-containing β phase observed in the quenched 5083 aluminum alloy. According to the spectral analysis results, the atomic percentage of Mg in the quenched 5083 aluminum alloy matrix was observed to be 4.73% (marked 5 in Figure 5b), which is far greater than that in the annealed 5083 aluminum alloy matrix with a Mg element content of 2.91% (marked 6 in Figure 5a). It can be deduced that there are many more solute Mg atoms in the matrix of quenched 5083 aluminum alloy sheet than in the matrix of the annealed 5083 aluminum alloy sheet. All of the 4.54% (wt. %) magnesium atoms can be dissolved in 5083 aluminum alloy matrix except a few remaining constituent particles when it is heated to 450 °C according to the Al-Mg binary phase diagram [21]. In addition, it is very difficult for the Mg-containing β precipitate to nucleate and grow from the α-Al solid solution in the quenched 5083 aluminum alloy sheet during water quenching treatment. Therefore, the Mg element is primarily supersaturated in the quenched 5083 aluminum alloy matrix and then forms Mg clusters during natural-aging treatment at room temperature. However, most of the Mg precipitated from the annealed 5083 aluminum alloy matrix as β precipitates because the cooling rate is slow enough during furnace cooling treatment. Overall, it can be concluded that the concentration of Mg atom in quenched 5083 aluminum alloy sheets is much higher than that in annealed 5083 aluminum alloy sheets, and the number of β precipitates in an annealed sheet is much higher than that in a quenched sheet.

**Figure 6:**
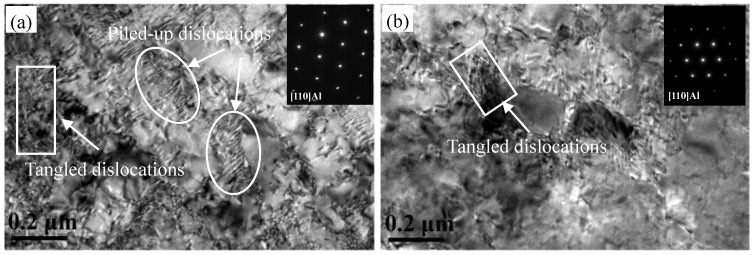
TEM images of (**a**) annealed and (**b**) quenched 5083 alloy sheets after tensile deformation.

Figure 6 shows TEM images of annealed and quenched 5083 aluminum alloy sheets after tensile deformation. Figure 6 indicates that dislocations increased largely after tensile deformation, many dislocations were blocked and tangled around the particles, and the dislocation pile-up zone in the annealed alloy (shown in Figure 6a) spread out over a larger area than that in the quenched alloy (shown in Figure 6b). In other words, the Mn-containing dispersoid and Mg-containing β phase particles are more resistant to dislocation penetration than Mg cluster. The dislocations almost follow the Orowan mechanism to bypass the Mn-containing dispersoid and Mg-containing β phase particles because the dispersoids and precipitates are relatively large, dislocation loops were subsequently left behind around the particles [21]. Thus, it can be considered that Mn-containing dispersoids and Mg-containing β phase particles both inhibit dislocation motion during tensile deformation.

### 3.2. Stress-Strain Curve and Strength of Two Heat Treatment Conditions of 5083 Aluminum Alloy Sheets

**Figure 7:**
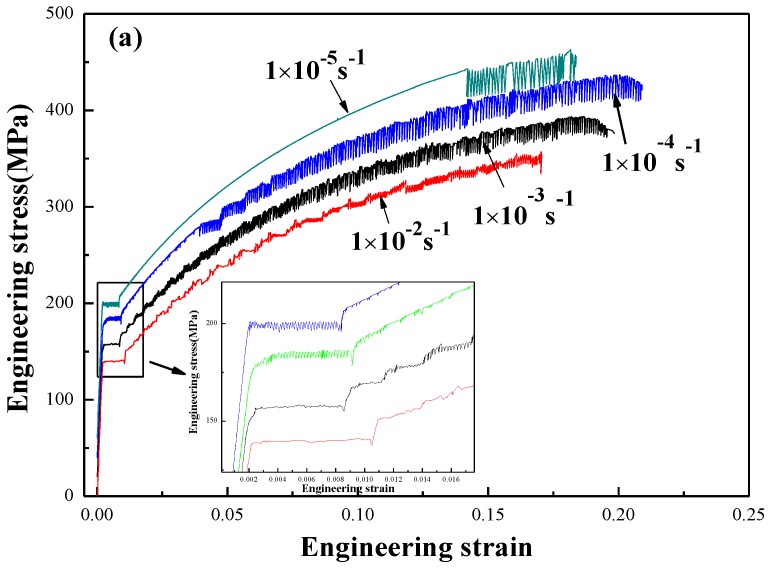

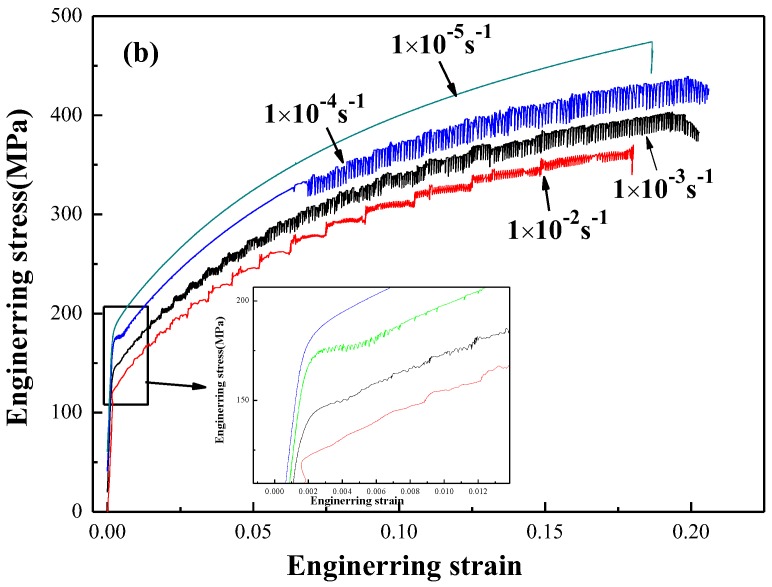
Engineering strain-engineering stress curves of (**a**) annealed and (**b**) quenched 5083 aluminum alloy sheets at various strain rates.

The engineering stress-engineering strain curves of annealed and quenched 5083 aluminum alloy sheets at various strain rates are shown in Figure 7. To display the curve clearly, the engineering stress-engineering strain curves at strain rates of 1 × 10^−3^ s^−1^, 1 × 10^−4^ s^−1^ and 1 × 10^−5^ s^−1^ were translated upward by 20 MPa, 40 MPa, and 60 MPa, respectively. Figure 7 shows that obvious serrated flow phenomena (also known as Portevin-Le Chatelier (PLC) effect) occurred in the engineering stress-engineering strain flow curves of all of the annealed alloy sheets (as shown in the Figure 7a) and most of the quenched alloy sheets, except for the specimens that are tensile deformed at a strain rate of 1 × 10^−5^ s^−1^ (as shown in the Figure 7b). 5083 aluminum alloy is a substitutional alloy, and the diffusion of solute Mg atom is the primary ingredient of the DSA. Hence, the PLC effect always occurs in this alloy [17,18,19,20]. The inhibition of the PLC effect, which was prevented from occurring in a quenched alloy sheet at a strain rate of 1 × 10^−5^ s^−1^, may insufficient effective stress caused by dynamic strain aging for dislocations to overcome the barrier of the solute atom. This observation was consistent with the results of 5456 aluminum alloy sheets not exhibiting the PLC effect in the tensile test at 60 ℃ at a strain rate of 5.4 × 10^−4^, as was studied by Fu et al. [24]. It also can be deduced that the diffusion velocity of high concentration Mg solute atoms in the aluminum matrix at room temperature can keep up with the velocity of moving dislocations during the whole tensile deformation process because of the sufficient amount of time for the Mg solute atoms to diffuse in the aluminum matrix when the strain rate was 1 × 10^−5^ s^−1^. In addition, it is notable that there was obvious yield plateau in all of the annealed 5083 aluminum alloy sheets at strains less than 0.012, and the width of the yield plateau increased as the strain rate increased, as shown in the partial enlargement in Figure 7a. In addition, no yield plateau was observed in quenched 5083 aluminum alloy sheets. Similar observations for Al-Mg alloys were reported in several studies, e.g., [25,26,27,28,29]. This phenomenon, called Piobert-Lüders bands, are most related to the interaction between movable dislocations and the segregation of diluted solute Mg atoms around the dislocation, called the Cottrell-Bilby atmosphere. As mentioned in Figure 5, the Mg concentration after annealing is much lower than that in quenched condition because more Mg atoms are consumed to form the β precipitates due to the lower cooling rate in annealing process than quenched condition. Therefore, there were much less diluted solute Mg atoms after annealing that can segregate at dislocations to form a Cottrell-Bilby atmosphere, making it easier for dislocations to pin and unpin, as well as gather collectively or self-organize, multiply, and move. At the initial stage of the tensile test, the deformation of the annealed sample is not uniform, and the specimen is divided into regions, known as Piobert-Lüders bands (Figure 7a). On the contrary, there were much more solute Mg atoms remaining in the Al matrix in the as-quenched condition. They can form the Mg clusters or GP zones in the early stage of natural-aging treatment, which can suppress the double cross-slip of screw dislocation and, therefore, suppress the Lüders effect or Lüders phenomenon (Figure 7b). In addition, the width of the yield plateau increased as the strain rate increased because the dislocation motion inertia increased as the strain rate increased.

According to the literature [30,31,32], the PLC deformation bands are categorized into type-A, type-B, and type-C by dynamic characteristics. From Figure 7, the alternation of PLC effects from type A to type B occurs in both annealed and quenched conditions with the decrease of strain rate and increase of strain. This can be attributed to the stronger interaction between mobile dislocations and particles, Mg clusters or solutes due to the increased density of mobile and immobile dislocations with the decrease of the strain rate or the increase of strain. 

**Figure 8:**
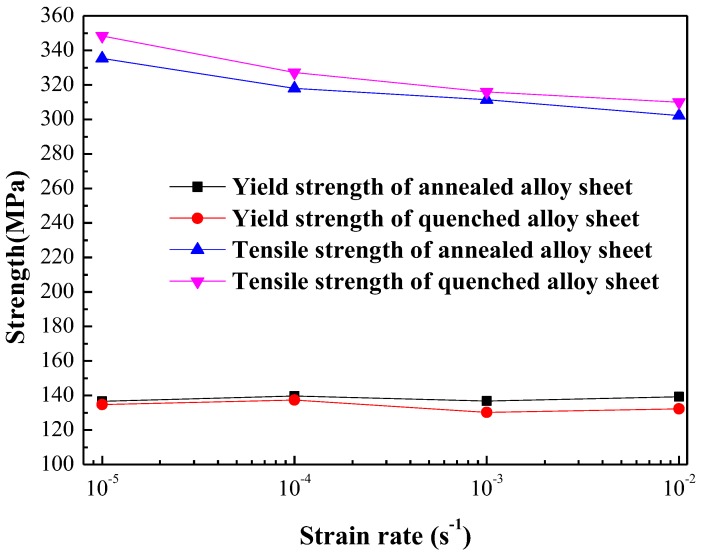
Relationship between strain rate and strength of annealed and quenched 5083 Al alloy sheets.

The strengths of annealed and quenched 5083 aluminum alloy sheets at various strain rates are shown in Figure 8. The yield strengths of annealed and quenched 5083 aluminum alloy sheets at different strain rates are found to be almost the same. However, the tensile strengths of quenched 5083 aluminum alloy sheets at different strain rates are slightly higher than those of annealed 5083 aluminum alloy sheets. The strain rate has almost no effect on the yield strength of both annealed and quenched 5083 aluminum alloy sheets, whereas the tensile strengths of annealed and quenched 5083 aluminum alloy sheets are decreased monotonously with increased strain rates. The interaction between moving dislocations and solute Mg atoms forms the atom atmosphere, which hinders dislocation from continuing to move and is the primary ingredient of not only the DSA and PLC effect, but also strain hardening behavior when the 5083 aluminum alloy sheets were tensile at room temperature. As mentioned above, the concentration of solute Mg atoms in quenched 5083 aluminum alloy is much higher than that in annealed alloy, and the degree of hindered dislocation motion and the work hardening ability in quenched alloys are higher than those in annealed alloys. These factors were the main cause of the tensile strength of quenched 5083 aluminum alloy sheets at different strain rates being slightly higher than those of annealed 5083 aluminum alloy sheets. The diffusion of Mg substitutional atoms could not keep up with the speed of moving dislocations when the tensile strain rate increased from 1 × 10^−5^ s^−1^ to 1 × 10^−2^ s^−1^ because of the lack of time for Mg substitutional atoms to form an atom atmosphere to block the moving dislocations, thereby decreasing the drag forces of the atom atmosphere. As a result, the tensile strength of the annealed and quenched 5083 aluminum alloy sheets decrease monotonously with the increase of the strain rates.

### 3.3. PLC Characteristics of Two Heat Treatment Conditions of 5083 Aluminum Alloy Sheets

**Figure 9:**
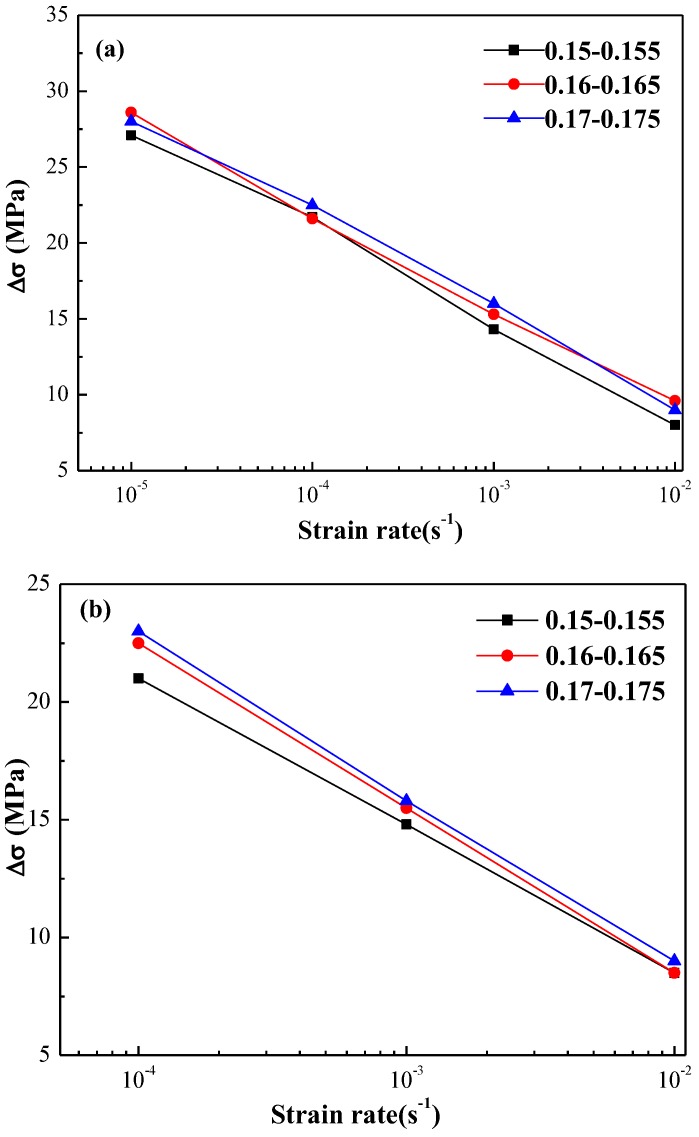
The relationship between the average serration magnitude and the strain rate in (**a**) annealed and (**b**) quenched 5083 Al alloy sheets.

As mentioned above, Figure 7 shows the typical stress-strain curves of the annealed and quenched 5083 aluminum alloy sheets deformed at strain rates from 1 × 10^−5^ s^−1^ to 1 × 10^−2^ s^−1^. The PLC effect was observed in both quenched and annealed 5083 aluminum alloy sheets, except the quenched 5083 aluminum alloy sheets tensile at 1 × 10^−5^ s^−1^. However, the statistical features of the serrations shown in Figure 7 are quite different from each other, with emphasis on the statistical features of the serrations. Figure 9 shows the relationship between average serration magnitude and the strain rate at different strain stages (0.150–0.155, 0.160–0.165, and 0.170–0.175) of annealed and quenched 5083 Al alloy sheets. The average serration magnitudes of the annealed and quenched alloy sheets are found to decrease linearly with the increase in the strain rate. Since the inertia and speed of moving dislocations increased as the strain rate increased, the waiting time that the moving dislocations require to overcome obstacles was shortened, and the number of solute Mg atoms segregate toward the dislocation to form atmosphere also decreased. Thus, the pinning strength of atmospheres is weakened. The applied stress required for dislocation to break free from the pinning decrease is manifested as a decrease in the magnitude of serration.

**Figure 10:**
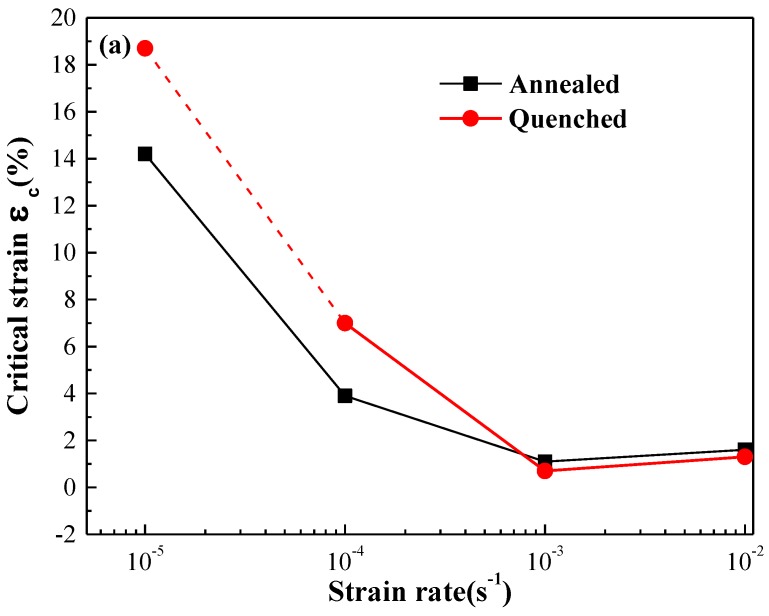

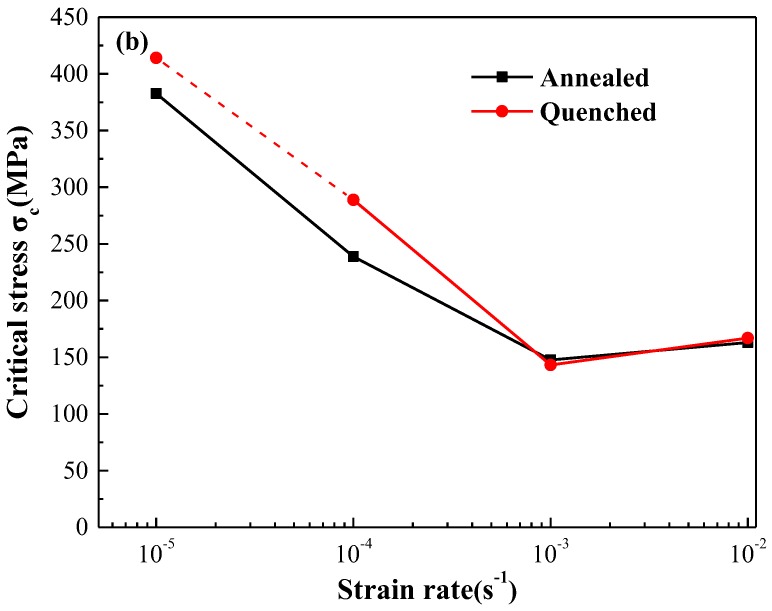
Relationship between strain rate and (**a**) critical strain (**b**) critical stress of the 5083 Al alloy after different heat treatments.

The relationship between critical strain, critical stress, and strain rate of annealed and quenched 5083 aluminum alloy sheets is shown in Figure 10. The critical strain ε_c_ and the critical stress σ_c_ are the strain value and stress value when jerky flow begins to appear in the stress-strain curve. Since there was no obvious PLC effect in the quenched specimen at a strain rate of 1 × 10^−5^ s^−1^, the fracture critical strain and fracture stress were considered the critical strain and critical stress of PLC effect, as shown in Figure 10 with dotted lines. The critical strain and critical stress of annealed and quenched alloy sheets were observed to both decreases first and then increase with the increasing strain rate, i.e., abnormal critical strain behavior occurs when the strain rate is less than 1 × 10^−3^ s^−1^, and normal critical strain behavior occurs when the strain rate exceeds 1 × 10^−3^ s^−1^. According to the “precipitation” model of Brechet and Estrin [33] the inverse behavior of critical strain only occurs in the alloy when the solute atom concentration is much higher than its equilibrium concentration at certain temperatures. In this work, the atomic percentage of Mg in the quenched and annealed 5083 aluminum alloy matrix was observed to be 4.73% (marked 5 in Figure 5b) and 2.91% (marked 6 in Figure 5a), respectively, which are much higher than the equilibrium concentration of Mg in Al matrix (1.9% at room temperature). This result agreed with the two critical strain models proposed by Fu [24,34]. When the strain rates are lower than 1 × 10^−3^ s^−1^, solute atoms have sufficient time to diffuse toward dislocations, and their diffusion rate can keep up with the movement of dislocations. As a result, the moving dislocations are always pinned by solute atoms at the early stage of plastic deformation, and the critical strain and critical stress at which the pinned dislocations break free from the solute atoms are determined by the first unpinning process. The number of substitutional Mg atoms around the moving dislocations decreased, and the pinning strength was weakened when the strain rates increased from 1 × 10^−5^ s^−1^ to 1 × 10^−3^ s^−1^ because the time for the solute atoms to diffuse toward dislocations is shortened. Therefore, the required applied stress for the moving dislocations to overcome the pinning of solute atoms decreased, which is the main reason that the critical strain and critical stress of both annealed and quenched 5083 aluminum alloys decreased when the strain rates increased form 1 × 10^−5^ s^−1^ to 1 × 10^−3^ s^−1^. However, the speed of moving dislocations is relatively high when the strain rates exceeds 1 × 10^−3^ s^−1^, solute atoms cannot keep up with the movement of dislocations and have nearly no pinning effect on the dislocation movement at the early stage of plastic deformation, and the critical strain and critical stress are determined by the first process of pinning by second phase particles, grain boundary, dislocation jog, and dislocation wall, etc. With the increase in the strain rate, the inertia of moving dislocations increased, and the obstacles that can pin the moving dislocations decreased, resulting in the critical strain and critical stress of both annealed and quenched 5083 aluminum alloys increasing with the increase in the strain rate when the strain rate exceeds 1 × 10^−3^ s^−1^.

In addition, the critical strain and critical stress of the quenched specimens are found to be higher than that of the annealed specimens when the strain rate is lower than 1 × 10^−3^ s^−1^, as seen in Figure 10. The critical strain depends on the first unpinning process when the strain rate is lower than 1 × 10^−3^ s^−1^ because the concentration of solute Mg atoms in the quenched sample is higher than that in the annealed specimens. As a result, the pinning strength of the solute atom group on movable dislocations is much greater, and dislocation depinning is more difficult in quenched specimens than in the annealed specimens. The movable dislocations require great activation energy and additional strain to break away from the pinning of the solute atoms. As the strain rate increases to 1 × 10^−3^ s^−1^ and 1 × 10^−2^ s^−1^, the critical strain of the annealed sample is slightly higher than that of the quenched sample. Since the critical strain of the 5083 aluminum alloy sheet depends on the first pinning process when the strain rate exceeds 1 × 10^−3^ s^−1^, the number of second-phase particles is higher and their distribution is more dispersed in the annealed specimens than in the quenched 5083 aluminum alloy specimens, resulting in increased hindrance to the movable dislocations at high strain rates, increased additional stress required for the depinning process, and higher critical strain in the annealed specimens than in the quenched 5083 aluminum alloy specimens.

**Figure 11:**
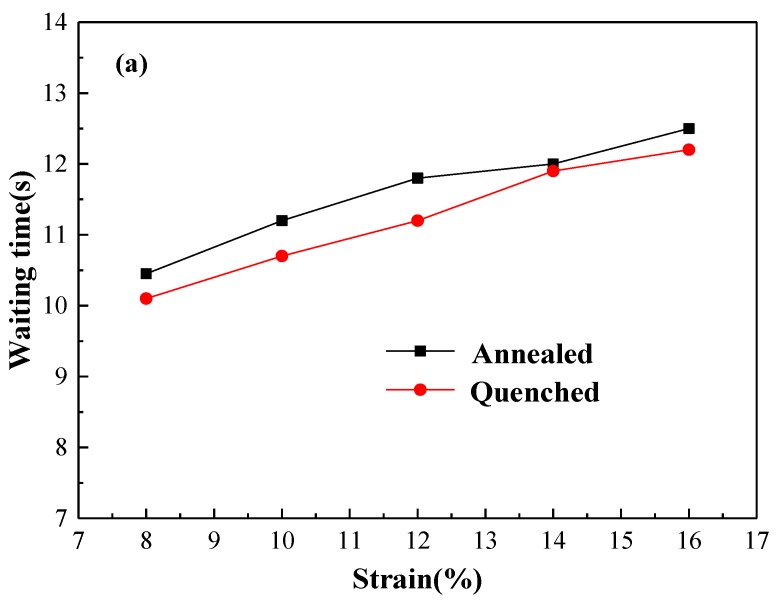

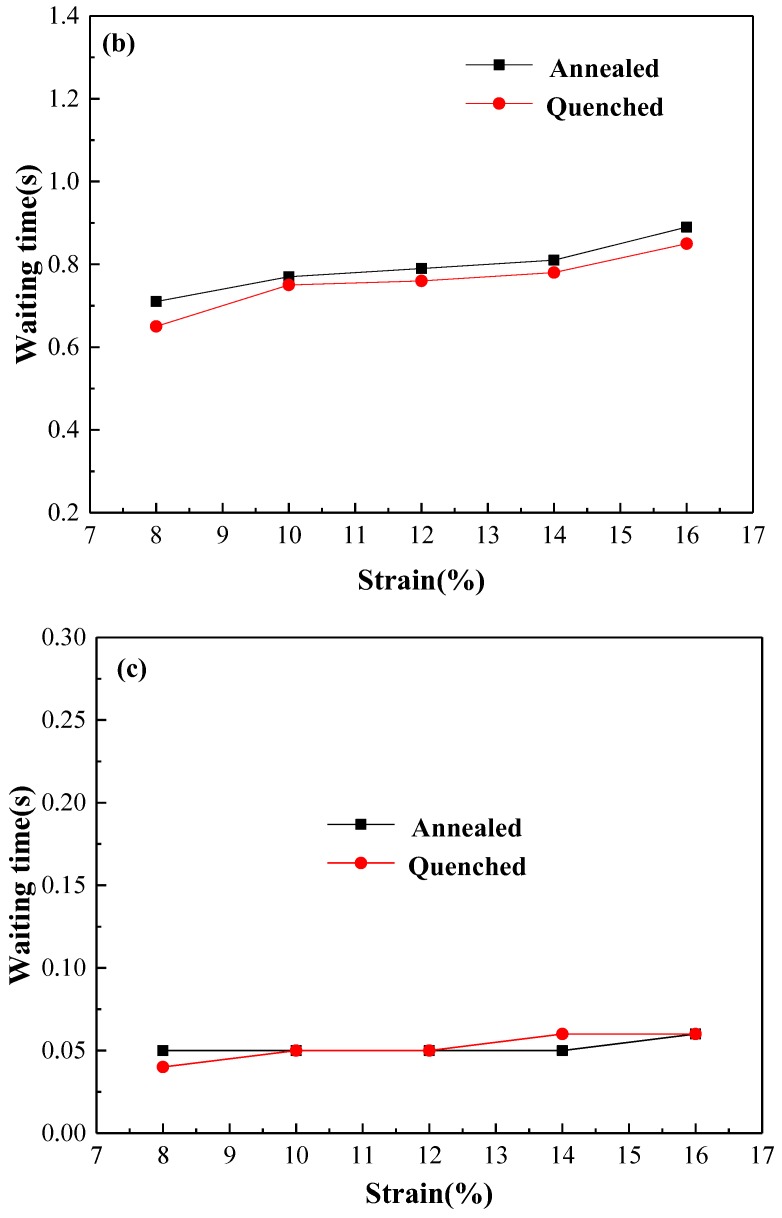
The waiting time (t_w_) of the annealed and quenched 5083 Al alloy sheets at different strain rates: (**a**) 1 × 10^−4^ s^−1^; (**b**) 1 × 10^−3^ s^−1^; and (**c**) 1 × 10^−2^ s^−1^.

Figure 11 shows the waiting time (t_w_) of the annealed and quenched 5083 Al alloy sheets at different strain rates. The waiting time of the annealed sample is found to be slightly longer than that of the quenched sample at a strain rate of 1 × 10^−4^ s^−1^ (as shown in Figure 11a), whereas the waiting times of the annealed and quenched samples are almost the same at strain rates of 1 × 10^−2^ s^−1^ and 1 × 10^−3^ s^−1^ (as shown in Figure 11b,c). The influence of the second-phase particles on the waiting time of annealed and quenched samples is not significant at high strain rates (1 × 10^−2^ s^−1^ and 1 × 10^−3^ s^−1^) because the speed of the moving dislocations is too fast. The hindering effect of the second-phase particles is significantly enhanced as the speed of the moving dislocations is relatively lower at a strain rate of 1 × 10^−4^ s^−1^. The higher amount of precipitates results in a greater hindering effect, leading to a longer period for movable dislocations to wait before continuing to move. In addition, the waiting time is shortened significantly from more than 10 s at a strain rate of 1 × 10^−4^ s^−1^ (as shown in the Figure 11a) to less than 0.05 s at a strain rate of 1 × 10^−2^ s^−1^ (as shown in the Figure 11c) when the strain rate increased. The velocity of moving dislocations increased as the strain rate increased, leading to the shortening of the waiting time (t_w_) that moving dislocations require to overcome the obstacles.

**Figure 12:**
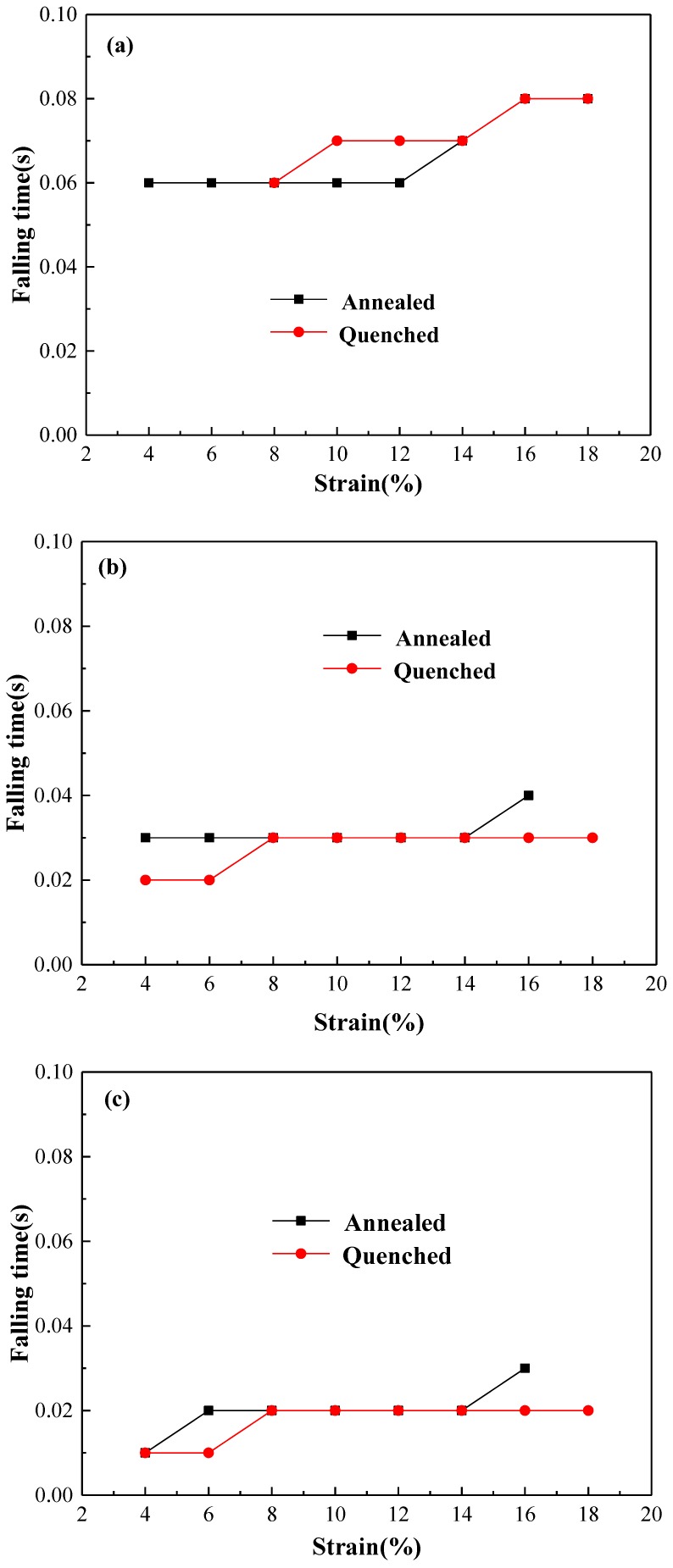
The falling time (t_d_) of the annealed and quenched 5083 Al alloy sheets at different strain rates: (**a**) 1 × 10^−4^ s^−1^; (**b**) 1 × 10^−3^ s^−1^; and (**c**) 1 × 10^−2^ s^−1^.

Figure 12 shows the falling time (t_d_) of the annealed and quenched 5083 Al alloy sheets at different strain rates. It can be seen that the falling times of the annealed and quenched specimens are almost the same at strain rates of 1 × 10^−4^ s^−1^, 1 × 10^−3^ s^−1^ and 1 × 10^−2^ s^−1^. The falling time (t_d_) is the length of time corresponding to a decrease in stress from a stress peak to the next adjacent stress valley, and this parameter corresponds to the process of the movable dislocations depinning from obstacles. Although the number of solute atoms and Mg-containing β precipitates are both different between annealed and quenched specimens, the total number of Mg atoms is the same in the annealed and quenched samples. The combined effect of solute atoms and β precipitates causes the same falling time of annealed and quenched samples at strain rates of 1 × 10^−2^ s^−1^, 1 × 10^−3^ s^−1^, and 1 × 10^−4^ s^−1^.

## 4. Conclusions

(1) The grain size, size, and dispersion of the remaining constituents and dispersoids in the annealed and quenched 5083 aluminum alloy sheets were approximately the same. However, the Mg atoms were mainly in the form of micron β precipitates after annealing but they present as Mg clusters in quenched condition.
(2) At both annealed and quenched condition, the applied strain rates have little effect on the yield strength, but the tensile strength decreases monotonously with the increase in strain rate. Meanwhile, the yield strength is similar for both conditions while the tensile strength of the quenched sheets is slightly higher than those annealed sheets. Obvious yield plateau or a serrated yielding phenomenon was observed in all of the annealed sheets at strains of less than 0.012, and its width increased as the strain rate increased. However, no serrated yielding phenomenon was observed in quenched sheets.
(3) The Portevin-Le Chatelier (PLC) effect occurred in the stress-strain flow curves of all of the annealed alloy sheets and most of the quenched alloy sheets, except for the specimens that are tensile deformed at a strain rate of 1 × 10^−5^ s^−1^.
(4) The average serration magnitude of the annealed and quenched sheets decreased linearly with increasing strain rate. The critical strain and critical stress of annealed and quenched alloy sheets both decrease first and then increase with increasing strain rate. When the strain rate is lower than 1 × 10^−4^ s^−1^, the critical strain stress of the quenched specimen are higher than those of the annealed specimen while they are is slightly lower when the strain rate increases to 1 × 10^−3^ s^−1^ and 1 × 10^−2^ s^−1^.
(5) The waiting time of the annealed specimen is slightly longer than that of the quenched specimen at a strain rate of 1 × 10^−4^ s^−1^, while they are similar at both annealed and quenched specimens at strain rates of 1 × 10^−2^ s^−1^ and 1 × 10^−3^ s^−1^. Additionally, the falling times of the annealed and quenched specimens are almost the same at the strain rates of 1 × 10^−4^ s^−1^, 1 × 10^−3^ s^−1^, and 1 × 10^−2^ s^−1^.

## References

[1] Huang K.T., Lui T.S., Chen L.H. (2011). Effect of microstructural feature on the tensile properties and vibration fracture resistance of friction stirred 5083 Alloy. J. Alloy. Compd..

[2] Bauri R., Yadav D., Kumar C.N.S., Balaji B. (2015). Tungsten particle reinforced Al 5083 composite with high strength and ductility. Mater. Sci. Eng. A.

[3] Newbery A.P., Nutt S.R., Lavernia E.J. (2006). Multi-scale Al 5083 for military vehicles with improved performance. Jom-Us.

[4] Benallal A., Berstad T., Borvik T., Clausen A.H., Hopperstad O.S. (2006). Dynamic strain aging and related instabilities: experimental, theoretical and numerical aspects. Eur. J. Mech. A/Solids.

[5] Chatterjee A., Sarkar A., Barat P., Mukherjee P., Gayathri N. (2009). Character of the deformation bands in the (A plus B) regime of the Portevin-Le Chatelier effect in Al–2.5%Mg alloy. Mater. Sci. Eng. A.

[6] Darowicki K., Orlikowski J. (2007). Impedance analysis of Portevin–Le Chatelier effect on aluminium alloy. Electrochim Acta.

[7] Shabadi R., Kumar S., Roven H.J., Dwarakadasa E.S. (2004). Characterisation of PLC band parameters using laser speckle technique. Mater. Sci. Eng. A.

[8] Sarkar A., Barat P., Mukherjee P. (2013). Investigation of Portevin-Le Chatelier Effect in Al-2.5 pct Mg Alloy with Different Microstructure. Metall. Mater. Trans. A.

[9] Cottrell A.H.S. (1954). Dislocations and Plastic flow in Crystals. Am. J. Phys..

[10] McCormick P.G. (1988). Theory of flow localisation due to dynamic strain ageing. Acta Metall..

[11] Mulford R.A., Kocks U.F. (1979). New observations on the mechanisms of dynamic strain aging and of jerky flow. Acta Metall..

[12] Sleeswyk A.W. (1958). Slow strain-hardening of ingot iron. Acta Metall..

[13] Van Den Beukel A., Kocks U.F. (1982). The strain dependence of static and dynamic strain-aging. Acta Metall..

[14] Springer F., Nortmann A., Schwink C. (1998). A study of basic processes characterizing dynamic strain ageing. Phys. Status Solidi A.

[15] Klose F.B., Ziegenbein A., Weldenmuller J., Neuhauser H., Hahner P. (2003). Portevin-LeChatelier effect in strain and stress controlled tensile tests. Comp. Mater. Sci..

[16] Cao P.T., Zhang Q.C., Fu S.H., Hu Q., Gao Y. (2010). Thermal analysis of serrated yielding in an Al-Mg alloy. Acta Phys. Sin..

[17] Reed J.M., Walter M.E. (2003). Observations of serration characteristics and acoustic emission during serrated flow of an Al-Mg alloy. Mater. Sci. Eng. A.

[18] Krishna K.S.V.B.R., Sekhar K.C., Tejas R., Krishna N.N., Sivaprasad K., Narayanasamy R., Venkateswarlu K. (2015). Effect of cryorolling on the mechanical properties of AA5083 alloy and the Portevin-Le Chatelier phenomenon. Mater. Des..

[19] Clausen A.H., Borvik T., Hopperstad O.S., Benallal A. (2004). Flow and fracture characteristics of aluminium alloy AA5083-H116 as function of strain rate, temperature and triaxiality. Mater. Sci. Eng. A.

[20] Joshi S.P., Eberl C., Cao B., Ramesh K.T., Hemker K.J. (2009). On the Occurrence of Portevin-Le ChA cent telier Instabilities in Ultrafine-Grained 5083 Aluminum Alloys. Exp. Mech..

[21] Hu Q., Zhang Q.C., Fu S.H., Cao P.T., Gong M. (2011). Effect of precipitation on Portevin-Le Chatelier effect in Al-Mg alloys. Acta Phys. Sin..

[22] Wen W., Morris J.G. (2004). The effect of cold rolling and annealing on the serrated yielding phenomenon of AA5182 aluminum alloy. Mater. Sci. Eng. A.

[23] Ratchev P., Verlinden B., Van Houtte P. (1995). Effect of preheat temperature on the orientation relationship of (Mn,Fe)Al6 precipitates in an AA 5182 Aluminium-Magnesium alloy. Acta Metall. Mater..

[24] Fu S.H., Cheng T., Zhang Q.C., Gao P.T., Hu Q. (2012). Study on two critical mechanisms of PLC effect of 5456 Al-Based Alloy. Acta Metall. Sin..

[25] Robinson J.M., Shaw M.P. (1994). Observations on deformation characteristics and microstructure in an Al-Mg alloy during serrated flow. Mater. Sci. Eng. A.

[26] Lloyd D.J., Court S.A., Gatenby K. (1997). Lüders elongation in Al-Mg alloy AA5182. Mater. Sci. Technol..

[27] Picu R.C., Vincze G., Ozturk F., Gracio J.J., Barlat F., Maniatty A.M. (2005). Strain rate sensitivity of the commercial aluminum alloy AA5182. Mater. Sci. Eng. A.

[28] Ohtani S., Inagaki H. (2002). Effect of heat treatment on yield point elongation and P-L effect in 5182 Al-Mg alloys. Mater. Sci. Forum.

[29] De Codes R.N., Hopperstad O.S., Engler O., Lademo O.G., Embury J.D., Benallal A. (2011). Spatial and Temporal Characteristics of Propagating Deformation Bands in AA5182 Alloy at Room Temperature. Metall. Mater. Trans. A.

[30] Xiang G.F., Zhang Q.C., Liu H.W., Jiang H.F., Wu X.P. (2006). Deformation measurements of three types of Portevin–Le Chatelier bands. Chin. Phys..

[31] Hu Q., Zhang Q.C., Cao P.T., Fu S.H. (2012). Thermal analyses and simulations of the type A and type B Portevin-Le Chatelier effects in an Al-Mg alloy. Acta Mater..

[32] Zhang Q.C., Jiang Z.Y., Jiang H.F., Chen Z.J., Wu X.P. (2005). On the propagation and pulsation of Portevin-Le Chatelier deformation bands: An experimental study with digital speckle pattern metrology. Int. J. Plasticity.

[33] Brechet Y., Estrin Y. (1995). On the influence of precipitation on the Portevin-Le Chatelier effect. Acta Metall. Mater..

[34] Fu S.H., Cheng T., Zhang Q.C., Hu Q., Cao P.T. (2012). Two mechanisms for the normal and inverse behaviors of the critical strain for the Portevin-Le Chatelier effect. Acta Mater..

